# Synergy between isobavachalcone and doxorubicin suppressed the progression of anaplastic thyroid cancer through ferroptosis activation

**DOI:** 10.1590/1414-431X2024e13679

**Published:** 2024-08-19

**Authors:** Shuai Lin, Hui Cai, Xuemei Song

**Affiliations:** 1 Department of Thyroid Breast Surgery, Affiliated Hospital of North Sichuan Medical College, Nanchong, Sichuan, China; 2 Department of Operating Room, Affiliated Hospital of North Sichuan Medical College, Nanchong, Sichuan, China

**Keywords:** Isobavachalcone, Doxorubicin, Anaplastic thyroid cancer, Ferroptosis, Reactive oxygen species

## Abstract

The objective of this study was to explore the effects and mechanisms of the combination of isobavachalcone (IBC) and doxorubicin (DOX) on the progression of anaplastic thyroid cancer (ATC). Cell viability of 8505C and CAL62 cells was observed by CCK-8 assay. Kits were used to detect the presence of reactive oxygen species (ROS), glutathione (GSH), malondialdehyde (MDA), and cellular iron. Protein expression of solute carrier family 7 member 11 (SLC7A11) and glutathione peroxidase 4 (GPX4) was detected using western blot, and CD31 was detected through immunofluorescence. Tumor xenograft models of 8505C cells were constructed to observe the effect of IBC and DOX on ATC growth *in vivo*. The co-administration of IBC and DOX exhibited a synergistic effect of suppressing the growth of 8505C and CAL62 cells. The concurrent use of IBC and DOX resulted in elevated iron, ROS, and MDA levels, while reducing GSH levels and protein expression of SLC7A11 and GPX4. However, the Fer-1 ferroptosis inhibitor effectively counteracted this effect. *In vitro* and *in vivo*, the inhibitory effect on ATC cell proliferation and tumor growth was significantly enhanced by the combination of IBC and DOX. The combination of IBC and DOX can inhibit the growth of ATC by activating ferroptosis, and might prove to be a potent chemotherapy protocol for addressing ATC.

## Introduction

Anaplastic thyroid cancer (ATC) is a rare but extremely aggressive solid malignancy that arises from thyroid follicular cells. Despite representing only 1% of thyroid cancers, ATC is the most aggressive tumor within this type of cancer and remains the primary cause of death among patients diagnosed with thyroid cancer. It is considered to be one of the deadliest solid malignant tumors in human beings (1,2). Upon diagnosis, the majority of ATC patients exhibit systemic illness. Even if surgery and external radiotherapy can locally control the disease, systemic therapy such as chemotherapy is also critical. Based on the 2018 National Comprehensive Cancer Network (NCCN) guidelines, the suggested chemotherapy treatments for ATC currently encompass the combination of paclitaxel and carboplatin, the combination of docetaxel and doxorubicin (DOX), or the use of paclitaxel or DOX as standalone agents (3). Among them, DOX is the most commonly used and is considered effective as a single drug, with about 30% of patients having partial remission when used alone (4). Despite being the most efficient chemotherapeutic medication for ATC treatment, the dosage of DOX is severely restricted because of its harmful adverse reactions. Furthermore, certain cancer cells exhibit resistance to DOX, which severely limits the clinical efficacy of DOX. Overcoming chemoresistance is an urgent problem to be solved.

In 2012, researchers first observed and recognized ferroptosis as a controlled, iron-dependent, non-apoptotic mechanism of cell death (5). The investigation reveals a strong association between ferroptosis, caused by an imbalance in reactive oxygen species (ROS), and the development of drug resistance in tumors. The exposure of tumor cells to chemotherapeutic medications can lead to a significant production of ROS, which poses considerable obstacles to the survival of tumor cells. Nevertheless, when tumor cells trigger a process to modify their metabolic environment, suppress ROS generation, and strengthen protection or tolerance against oxidative stress, this leads to the development of drug resistance (6). As a new type of programmed cell death, ROS-induced ferroptosis helps inhibit tumor growth and increase chemosensitivity, targeting the pathway that regulates ferroptosis in tumor cells. The use of ferroptosis-inducing drugs that have shown great potential in tumor treatment of tumors is an emerging cancer therapy (7,8). Therefore, it is urgent to develop more efficient inducers of ferroptosis in ATC cells with low toxicity and few side effects in normal cells.

Isobavachalcone (IBC) is an active ingredient extracted from the fruit of the plant psoralen, which belongs to natural chalcone-type small molecule compounds. It is a class of isopentenyl chalcone derivative, which chemical formula is C_20_H_20_O_4_. Research has indicated that IBC possesses diverse pharmacological properties including antitumor, hemostatic, antibacterial, and immune-enhancing effects, which has led to a growing interest in its clinical applications. IBC, as a natural compound with high efficiency, low toxicity, and economical cytotoxicity, has obvious inhibitory effects on various malignant tumors (9).

In this study, we used IBC combined with DOX to treat human ATC (8505C and CAL62) cell lines. IBC was found to activate ferroptosis in ATC cells through ROS production.

## Material and Methods

### Cell culture and reagents

The Tongpai Bio Technology Co., Ltd. (China) provided the Human ATC cell lines (8505C and CAL62), which were cultivated in the DMEM (12491015, Gibco, USA) medium containing 15% FBS (10099158, Gibco) at a temperature of 37°C in a humid environment with 5% CO_2_. IBC (I422520), DOX (D344975), and Ferrostatin-1 (Fer-1, F408509) were purchased from Aladdin Reagent Co., Ltd. (China). The CCK-8 (C0038), EDU (C0075S), and MDA (S0131S) kits were purchased from Beyotime (China). The iron ion detection kit (ab83366) was purchased from Abcam (China). The GSH kit (A006-2-21) was bought from NanJing JianCheng Bioengineering Institute (China). The ROS kit (E-BC-K138-F) was bought from Elabscience Biotechnology Co., Ltd. (China). SLC7A11 (DF12509), GPX4 (DF6701), GAPDH (AF7021), and CD31 (AF6191) antibodies were purchased from Affinity (China).

### Cell grouping

The grouping for 8505C cell line was: control group: PBS; DOX group: 0.491 μM DOX; IBC group: 94.98 μM IBC; DOX+IBC group: 0.491 μM DOX+50 μM IBC; DOX+IBC+Fer-1 group: 0.491 μM DOX+50 μM IBC+1.0 μM Fer-1; Fer-1 group: 1.0 μM Fer-1.

The grouping for CAL62 cell line was: control: PBS; DOX group: 0.673 μM DOX; IBC group: 152.5 μM IBC; DOX+IBC group: 0.673 μM DOX+100 μM IBC; DOX+IBC+Fer-1 group: 0.673 μM DOX+100 μM IBC +1.0 μM Fer-1. Fer-1 group: 1.0 μM Fer-1.

All cells were treated for 48 h and collected for subsequent assays.

### Cell viability assay

Cell viability was assessed by the CCK-8 assay. Cells were grown on 96-well plates with each well containing 5,000 cells. IBC and DOX were added at the concentrations indicated after 48 h. The microplate reader (Multiskan MK3, Thermo Scientific, USA) was used to measure the absorbance at 450 nm.

### EDU staining assay

A total of 1.5×10^4^ cells per well were seeded onto 96-well plates. After treatment with the drug for 48 h, EDU reagent (1:1000 dilution) was added to each well for 2 h. Then, 4% paraformaldehyde was used to fix the cells, and fluorescent dye and DAPI were used to stain cells. The samples were then photographed and analyzed using a fluorescence microscope (DMI3000B, Leica, Germany).

### Determination of ROS generation

The concentration of DCFH-DA was diluted to 10 μM/L using serum-free medium in a ratio of 1:1000. After removal of the medium, 1 mL of diluted DCFH-DA was introduced into each well, and then incubated at 37°C for 20 min in a cell incubator. The cells were then rinsed three times with serum-free cell culture medium to effectively eliminate any excess DCFH-DA. Subsequently, detection was carried out using a flow meter (BD, FACSCalibur, China).

### Determination of GSH and MDA

GSH and MDA levels were determined with GSH and MDA kits. After treatment with the drug for 48 h, the cells were obtained and rinsed two times using PBS. Isotonic PBS buffer was added to resuspend the cells followed by vortexing (2000 rpm, 10 s), and then the cells were disrupted using sonication. A 0.1 mL portion of the disrupted cell suspension was taken and mixed with 0.1 mL of working reagent for 5 min. For tumor tissues, 0.1 g of tissue of each group was added with 1 mL of extracting solution to homogenize in ice bath, it was centrifuged at 8000 *g* at 4°C for 10 min, and the supernatant was then placed on ice for measurement following the manufacturer's protocol. A microplate reader (Multiskan MK3, Thermo Scientific) was used to measure the absorbance under 450 nm (GSH) and 532 nm (MDA).

### Iron level detection

The iron assay was conducted in accordance with the manufacturer's protocol. In summary, the specimens were subjected to incubation with an iron reducer solution (ab83366, Abcam) at a temperature of 25°C for a duration of 30 min, followed by further incubation for 60 min with an iron probe at the same temperature. Subsequently, the level of iron was measured using a microplate reader at a wavelength of 593 nm.

### Western blot detection

Following transfer and blocking, the primary antibody (SLC7A11 antibody 1:1000, GPX4 antibody 1:1000, and GAPDH 1:3000) were placed in the PVDF membrane. The samples were incubated for an entire night at a temperature of 4°C. Afterwards, the membrane was treated with HRP-conjugated secondary antibody and incubated at room temperature for 1 h. The premixed ECL luminescent substrate was then added dropwise, and the results were captured using a chemiluminescence imaging system (GeneGnome Series 3600, China).

### *In vivo* tumor xenograft study

Six groups were formed by randomly assigning female BALB/c nude mice, aged 6-8 weeks (18-20 g), provided by Changzhou Kavins Experimental Animal Co., Ltd. (China). The mice were provided with standard chow and water on a 12-h light/dark cycle under specific-pathogen-free conditions. The left axillary skin of nude mice was sterilized, and 100 μL of a suspension of 2×10^6^/100 μL 8505C cells was injected subcutaneously into one BALB/c nude mouse. When the tumor size reached approximately 100 mm^3^, the mice were intraperitoneally injected with DOX (5 mg/kg), IBC (50 mg/kg), the combination of DOX (5 mg/kg) and IBC (50 mg/kg), the combination of DOX (5 mg/kg), IBC (50 mg/kg), and Fer-1 (10 mg/kg), or Fer-1 alone treatment (10 mg/kg). All the drugs were injected once a week for 4 weeks. After group treatment, tumor volume was monitored every 7 days, and the tumor growth curve was drawn. Following a month of therapy, the mice were euthanized, and the tumors excised and weighed. Tumor size was measured using Vernier calipers, and tumor volume was calculated. The fresh tumor tissue was preserved at a temperature of -80°C. All animal handling and experimental procedures were approved by the Medical Ethics Committee of Affiliated Hospital of North Sichuan Medical College (Approval No. 2023ER408-1) and in accordance with the Declaration of Helsinki of the World Medical Association.

### Immunofluorescence assay to detect CD31 expression

Tumor tissues obtained from the mouse xenograft model were fixed using 4% paraformaldehyde for 48 h at 4°C. The tumor sections were cut at a thickness of 10 µm using a freezing microtome. The sections were blocked and permeabilized in 5% goat serum solution containing 0.3% Triton X-100 for 2 h at room temperature. The sections were incubated with a rabbit anti-CD31 primary antibody (1:500) overnight at 4°C. A diluent for the secondary antibody, goat anti-rabbit IgG (H+L) FITC, was prepared and incubated at 37°C for 3 h after being diluted at a ratio of 1:500. Nuclei were counterstained with DAPI, and then the stained sections were observed using a fluorescence microscope (Leica, DMI3000B).

### Statistical analysis

Statistical analysis was performed using Prism 8.0.1 GraphPad software (USA). Data are reported as means±SD. One-way ANOVA was utilized for multiple-group comparisons, while the *t*-test was used to compare two groups. P<0.05 was considered statistically significant.

## Results

### IBC synergized with DOX to inhibit ATC cell viability

**Figure 1:**
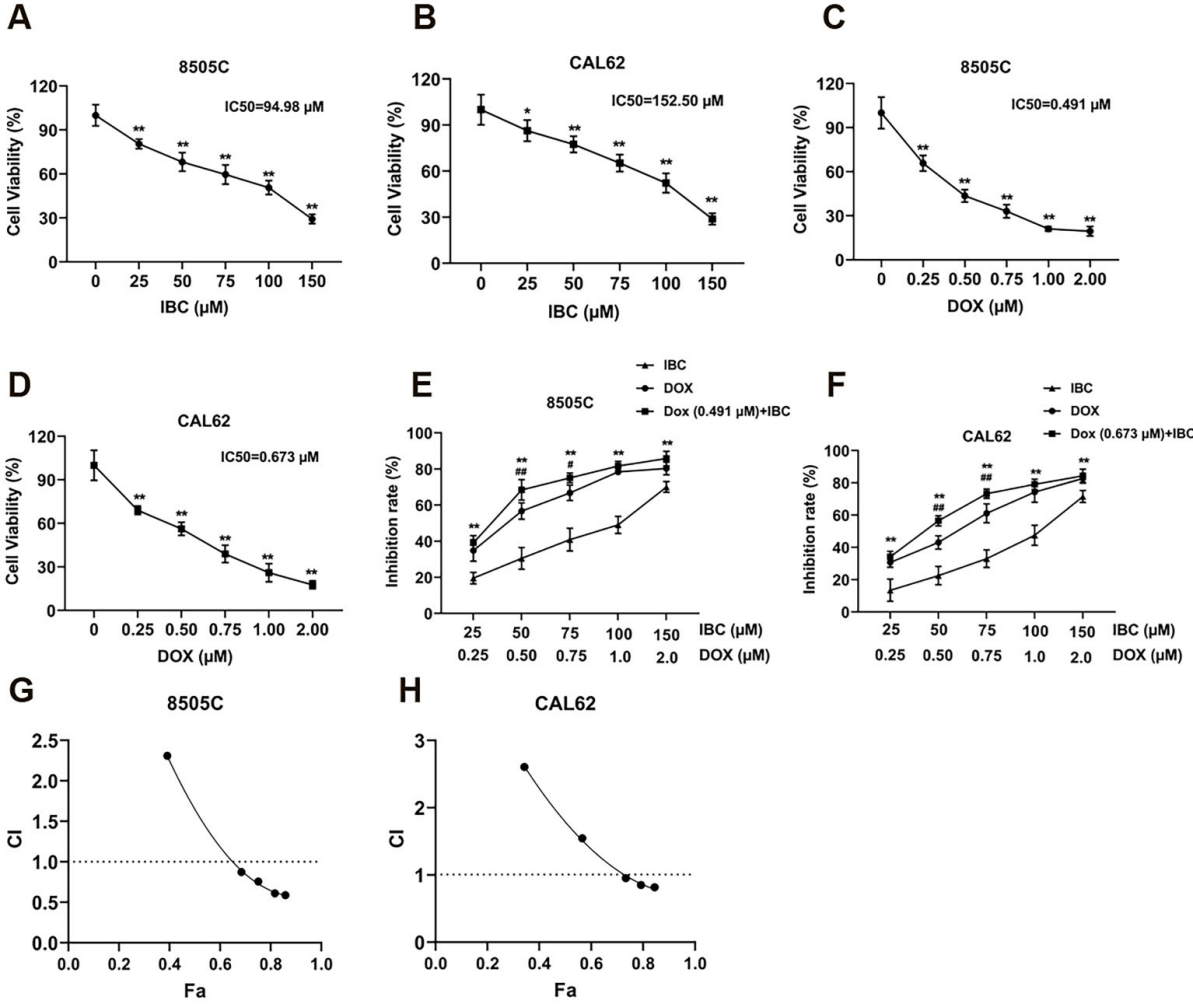
Isobavachalcone (IBC) synergizes with doxorubicin (DOX) to inhibit anaplastic thyroid cancer (ATC) cell viability. **A** and **B**, Cell viability detected by CCK-8 in 8505C and CAL62 cells after treatment with different concentrations of IBC. **C** and **D**, Cell viability after treatment with indicated concentrations of DOX. **E** and **F**, Cell inhibition rate in 8505C (**E**) and CAL62 (**F**) cells after co-administration with different concentrations of IBC and DOX [half-maximum inhibitory concentrations (IC50) concentration] for 48 h. Data are reported as mean and SD. **P<0.01 *vs* IBC alone group; ^#^P<0.05, ^##^P<0.01 *vs* DOX alone group (ANOVA). **G** and **H**, Drug combination index (CI) of DOX and IBC in 8505C and CAL62 cells was calculated via the Chou-Talalay method using CompuSyn software. All experiments were carried out at least three times.

The 8505C and CAL62 cells were treated with 0, 25, 50, 75, 100, and 150 μM of IBC and 0, 0.25, 0.5, 0.75, 1.00, and 2.00 μM of DOX for 48 h, respectively. CCK-8 assay was applied to examine cell viability. The viability of ATC cells was significantly decreased in response to increasing IBC concentrations. The half-maximum inhibitory concentrations (IC50) of IBC in 8505C and CAL62 cells were 94.98 and 152.5 μM, respectively (Figure 1A and B). Cell viability was attenuated with DOX treatment, and the IC50s of DOX in 8505C and CAL62 cells were 0.491 and 0.673 μM, respectively (Figure 1C and D). IBC and DOX alone efficiently reduced the viability of ATC cells. Co-administration of 8505C and CAL62 cells was carried out according to DOX IC50 (0.491, 0.673 μM, respectively) and IBC gradient concentrations. CCK8 detection and calculation of cell inhibition rate at each concentration in 8505C cells is shown in Figure 1E and in CAL62 cells in Figure 1F. Based on the results of Figure 1E and F, the drug combination index (CI) of DOX and IBC in 8505C and CAL62 cells was calculated via the Chou-Talalay method (10) using CompuSyn software (ComboSyn, Inc., USA) (Figure 1G-H and Table 1). CI values less than 1.0, 1.0, and greater than 1.0 indicate synergy, additive, and antagonism between compounds, respectively (11). These results revealed that the combination of IBC (50, 75, 100, and 150 μM) and DOX (0.491 μM) in 8505C cells and the combination of IBC (75, 100, and 150 μM) and DOX (0.673 μM) in CAL62 cells had a synergistic anti-tumor effect.

**Table 1:** Combination index (CI) values at different doses detected via the Chou-Talalay method using CompuSyn software.

Cells	IBC (µM)	DOX (µM)	Effect	CI
8505C	25.0	0.491	0.391	2.309
	50.0	0.491	0.684	0.875
	75.0	0.491	0.750	0.757
	100.0	0.491	0.816	0.611
	150.0	0.491	0.858	0.589
CAL62	25.0	0.673	0.342	2.607
	50.0	0.673	0.565	1.546
	75.0	0.673	0.733	0.954
	100.0	0.673	0.791	0.850
	150.0	0.673	0.844	0.818

IBC: Isobavachalcone; DOX: Doxorubicin. CI values less than 1.0, 1.0, and greater than 1.0 indicate synergism, additivity, and antagonism, respectively.

### IBC combined with DOX regulated ferroptosis-associated GSH decrease, MDA increase, and ROS accumulation of ATC cells

**Figure 2:**
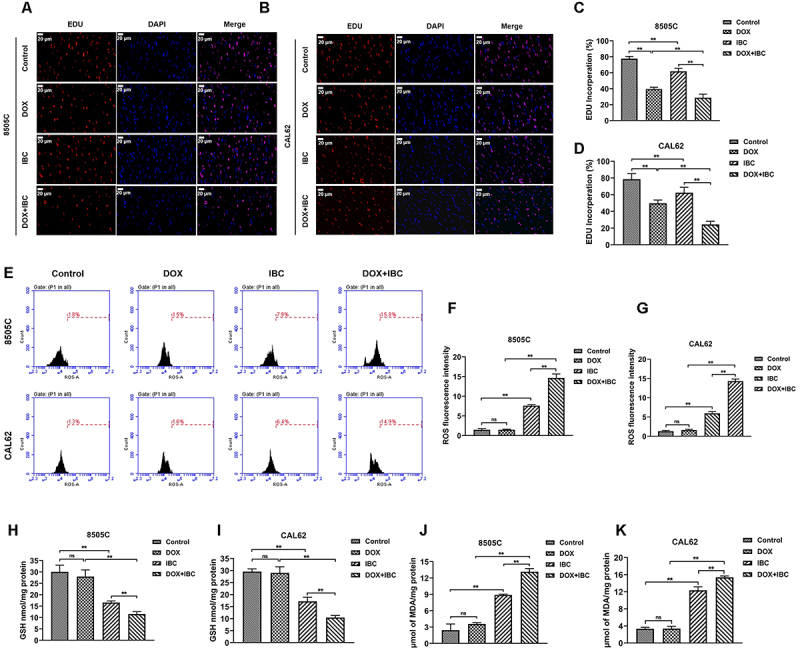
The combination of isobavachalcone (IBC) and doxorubicin (DOX) regulated ferroptosis-associated glutathione (GSH) decrease, malondialdehyde (MDA) increase, and reactive oxygen species (ROS) accumulation of anaplastic thyroid cancer (ATC) cells. **A** and **B**, EDU staining of 8050C and CAL62 cells treated with IBC, DOX, or their co-administration after treatment for 48 h. Scale bar 20 μm. **C** and **D**, Quantification analysis of EDU staining. **E**-**G**, Cellular ROS was analyzed with the DCFH-DA kit after treatment for 48 h. **H** and **I**, Cellular GSH was analyzed with the GSH kit after treatment for 48 h. **J** and **K**, MDA levels were analyzed using MDA kit after treatment for 48 h. Data are reported as mean and SD. **P<0.01; ns: not significant (ANOVA). All experiments were carried out at least three times.

As shown in Figure 2A-D, the results of EDU staining indicated that DOX and IBC alone significantly inhibited cell proliferation, and the reduction of cell proliferation with co-administration of DOX and IBC was greater compared to DOX or IBC alone. Compared with the control group, DOX alone had no significant changes in ROS (Figure 2E-G), GSH (Figure 2H and I), and MDA (Figure 2J and K) levels, while IBC alone significantly increased ROS and MDA levels and decreased GSH levels. Compared with the DOX alone group or the IBC group, the levels of ROS and MDA were further increased, and the level of GSH was further decreased in the DOX+IBC combined group.

### IBC combined with DOX regulated the intracellular iron level and ferroptosis-related proteins

**Figure 3:**
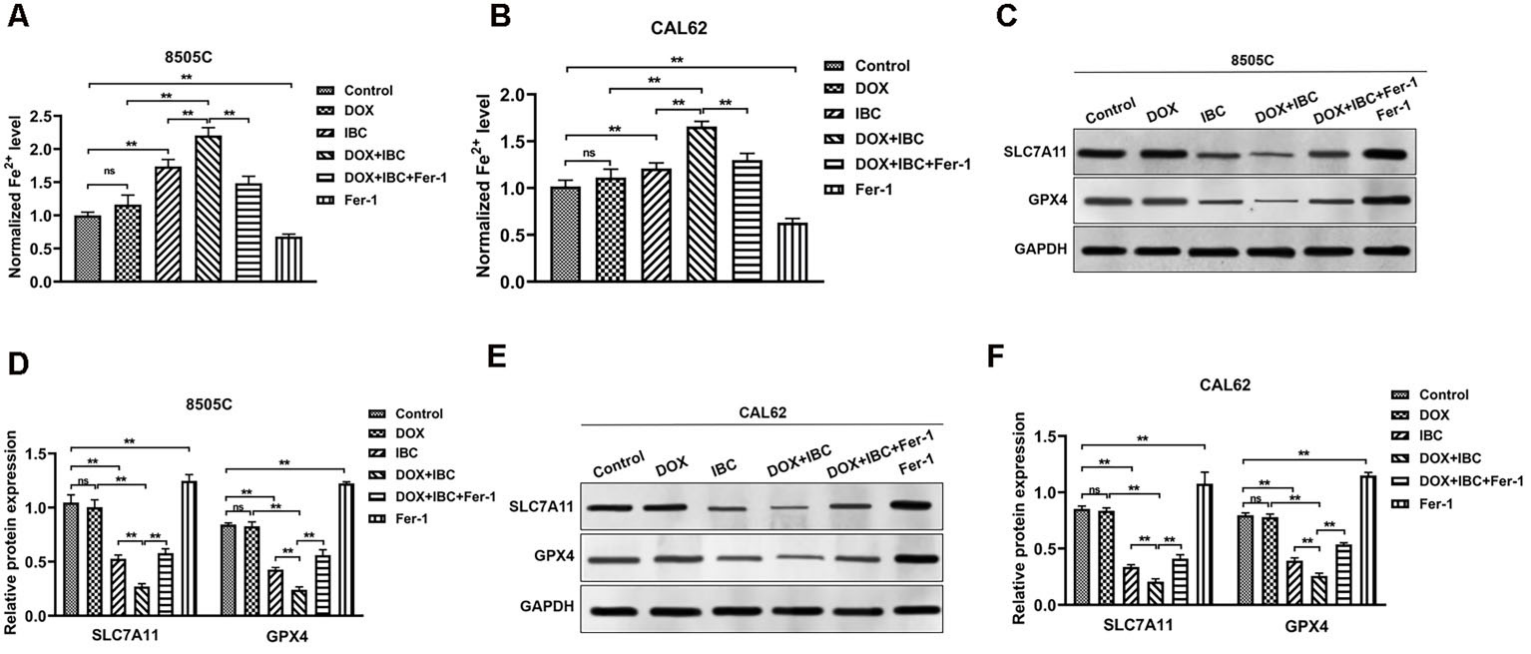
Isobavachalcone (IBC) combined with doxorubicin (DOX) regulated the intracellular iron level and of ferroptosis-related proteins. **A** and **B**, Cellular iron levels were measured using the iron ion detection kit after treatment for 48 h. **C**-**F**, Western blot detection and quantification analysis of the SLC7A11 and GPX4 protein in 8505C (**C** and **D**) and CAL62 cells (**E** and **F**) after treatment for 48 h. Data are reported as mean and SD. **P<0.01; ns: not significant (ANOVA). All experiments were carried out at least three times.

DOX alone had no significant changes in iron level, while IBC alone significantly increased iron levels in 8505C and CAL62 cells (Figure 3A and B). Furthermore, the addition of Fer-1 effectively reduced iron levels. Moreover, western blot was applied to examine the expression of the ferroptosis-related SLC7A11 and GPX4 proteins in 8505C and CAL62 cells (Figure 3C-F). The results indicated that the expression levels of the SLC7A11 and GPX4 proteins did not change significantly in DOX alone compared to the control group, while IBC alone significantly decreased the expression levels of the SLC7A11 and GPX4 proteins. Compared to the DOX group or the IBC group, the expression levels of the SLC7A11 and GPX4 proteins in the DOX+IBC combined group were further reduced. Compared to the DOX+IBC combined group, the addition of Fer-1 significantly increased the expression levels of the SLC7A11 and GPX4 proteins. Moreover, treatment with Fer-1 alone significantly inhibited the iron levels and promoted the expression of SLC7A11 and GPX4 in 8505C and CAL62 cells. The results suggested that IBC may induce tumor suppression by regulating the ferroptosis pathway.

### IBC combined with DOX suppressed ATC tumor growth and angiogenesis *in vivo*

**Figure 4:**
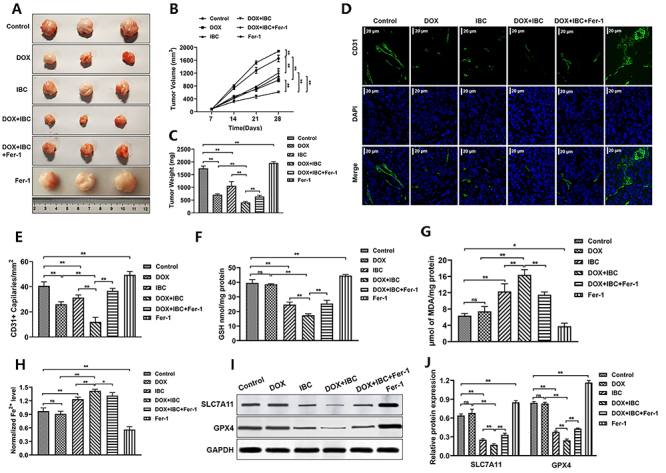
Isobavachalcone (IBC) combined with doxorubicin (DOX) suppressed anaplastic thyroid cancer (ATC) tumor growth and angiogenesis *in vivo*. **A**, Tumor tissues. **B**, Tumor volume. **C**, Tumor weight. **D** and **E**, Immunofluorescence staining of CD31 in tumor tissues and quantitative analysis. CD31 (green) indicates blood vessels; DAPI (blue) indicates nuclei. Scale bar 20 μm. n=3. **F**-**H**, The levels of glutathione (GSH) (**F**), malondialdehyde (MDA) (**G**), and cellular iron (**H**) were detected using the correspondent kits, n=3. **I** and **J**, Western blot detection and statistical results of the SLC7A11 and GPX4 protein *in vivo*. Data are reported as mean and SD. *P<0.05, **P<0.01; ns: not significant (ANOVA). All experiments were carried out at least three times.

CD31 is also known as platelet endothelial cell adhesion molecule (platelet endothelial cell adhesion molecule-1, PECAM-1/CD31). In immunohistochemistry, CD31 is used to assess tumor angiogenesis, which is a novel microvascular marker (12). Tumor tissue was collected for body weight detection and CD31 immunofluorescence staining to detect blood vessel density in nude mice carrying 8505C ATC xenografts. The results showed that, compared with the control group, the single DOX or IBC treatment significantly reduced tumor volume (Figure 4A and B) and weight (Figure 4C), and significantly decreased the tumor micro-angiogenesis rate (Figure 4D and E). Compared to the DOX or IBC alone group, tumor volume and weight in the DOX+IBC group were further reduced, and tumor micro-angiogenesis rate was further significantly reduced. Moreover, compared with the DOX+IBC combined administration group, the addition of Fer-1 significantly promoted tumor growth and tumor angiogenesis.

In addition, compared to the control group, DOX alone did not show significant changes in GSH (Figure 4F), MDA (Figure 4G), iron (Figure 4H), and in expressions of SLC7A11 and GPX4 (Figure 4I and J), while IBC alone significantly increased ROS, MDA, and cellular iron levels, but decreased the concentrations of GSH, SLC7A11, and GPX4. Compared with the DOX group or the IBC group, the levels of ROS, MDA, and cellular iron were further increased, but the GSH, SLC7A11 and GPX4 concentrations were further decreased in the DOX+IBC combined group. Compared to the DOX+IBC group, the addition of Fer-1 significantly decreased ROS, MDA, and cellular iron levels and increased the levels of GSH, SLC7A11, and GPX4. Furthermore, the Fer-1 alone treatment significantly promoted tumor growth and tumor micro-angiogenesis rate *in vivo* compared to the control group. Besides, the Fer-1 alone treatment increased the levels of GSH and decreased MDA and iron levels. The data from *the vivo* experiments further confirmed that IBC may affect the tumor suppressor effects by regulating the ferroptosis pathway.

## Discussion

ATC remains a clinical therapeutic challenge due to its dedifferentiated phenotype and highly aggressive nature. The latest research shows that targeted therapy drugs such as sorafenib, imatinib, etc. can control the process of ATC (13), and Phase I-II studies of immunotherapy drugs such as anti-spartalizumab (an anti-PD1 monoclonal antibody) show some efficacy in ATC (14). However, they are all in the early stages of clinical trials such as Phase I and Phase II, and relatively few clinical ATC patients are involved. Lin et al. (15) discovered that the collaboration between GSK-J4 and doxorubicin could potentially create a powerful chemotherapy treatment for ATC with KRAS mutations. Hence, chemotherapy medications remain a crucial approach for the systemic management of ATC. DOX has demonstrated its efficacy as the foremost clinical cytotoxic medication for ATC therapy in recent years. Nevertheless, when used alone, the effectiveness of this treatment is restricted due to issues like acquired resistance (4). This study showcased the effective inhibition of ATC cell growth and xenograft models of ATC tumor tissue through the synergistic combination of IBC and DOX.

In this study, we evaluated IBC, a sensitive anticancer active ingredient from traditional Chinese medicine and natural plants and animals, which can induce neuroblastoma cell apoptosis, but has no significant effect on cerebellar granulosa cells in a mouse model *in vitro* (16). Jing et al. (17) found that IBC promotes the apoptosis of tumor cells (ovarian carcinoma cell OVCAR-8, prostate carcinoma cell PC3, breast carcinoma cell MCF-7, and lung carcinoma cell A549), while having no toxic side effects on normal cells. The results of another study also showed that while IBC had a killing effect on tumor cell lines OVCAR8, PC3, A549, and MCF7, it did not have an obvious killing effect on normal cells, further proving that IBC can be used as a low-toxicity and highly effective antitumor drug in clinical application (18). Further research on the pharmacological mechanism of IBC shows that in addition to promoting tumor cell apoptosis, inhibiting tumor cell metastasis, and other biological activities, IBC can also overcome tumor drug resistance (19). IBC is also described as a potent inhibitor of the Akt signaling pathway (20). Recent studies have shown that inhibition of PI3K/Akt/mTOR signaling could be a promising molecular target for thyroid cancer therapy (21). The roles and underlying mechanisms of IBC in thyroid cancer therapy deserve in-depth investigation. In this study, we found that IBC could inhibit ATC cell growth. However, when IBC and DOX were combined, we found a synergistic effect in inhibiting the proliferation of 8505C and CAL62 cells.

There is growing evidence suggesting that enhancing ferroptosis could be a viable strategy to reduce drug resistance in cancer treatment (22,23). Research has shown that ROS have a significant impact on the control of chemosensitivity. The process of tumor cells to alter their metabolic surroundings and hinder the creation of ROS leads to the development of drug resistance (24). Roh et al. (25) discovered that erastin, a substance that triggers ferroptosis, can hinder the growth of cells in head and neck squamous cell carcinoma, enhance the buildup of ROS, and induce ferroptosis. More importantly, the silencing System Xc^−^ can increase the sensitivity of cisplatin-resistant cells to cisplatin by inducing ferroptosis. Additional research has revealed that erastin has the potential to greatly augment the effectiveness of DOX as a primary chemotherapy drug in HL60 cells (human promyelocytic leukemia cells). Moreover, the induction of ferroptosis has the ability to overcome drug resistance in acute myeloid leukemia cells, as indicated by previous studies (26,27). Therefore, studies suggest that tumor cell growth can be controlled by regulating the ferroptosis mechanism and by the use of related inducers and inhibitors. Ferroptosis can be used as a new target for tumor suppression and its inducers can be used as adjuvant drugs for chemotherapy in the preclinical and clinical treatment of tumors (7,27). This study confirmed that the combination of IBC and DOX effectively decreased the GSH level and elevated the ROS and MDA levels in both 8505C and CAL62 cells, both *in vivo* and *in vitro*.

### Conclusion

In summary, we found that IBC induced ferroptosis in ATC cells by activating ROS, which helps to inhibit ATC growth and increase chemosensitivity to DOX, laying a theoretical foundation for the future treatment of ATC. The detailed molecular mechanism of this synergy remains to be further investigated. However, it is worthy of research, development, and utilization as a novel natural high-efficiency and low-toxicity small molecule inhibitor in combination with doxorubicin in the treatment of ATC.

## References

[1] Rao SN, Smallridge RC (2023). Anaplastic thyroid cancer: an update. Best Pract Res Clin Endocrinol Metab.

[2] Ferrari SM, Elia G, Ragusa F, Ruffilli I, La Motta C, Paparo SR (2020). Novel treatments for anaplastic thyroid carcinoma. Gland Surg.

[3] Haddad RI, Nasr C, Bischoff L, Busaidy NL, Byrd D, Callender G (2018). NCCN Guidelines Insights: Thyroid Carcinoma, Version 2.2018. J Natl Compr Canc Netw.

[4] Miccoli P, Materazzi G, Antonelli A, Panicucci E, Frustaci G, Berti P (2007). New trends in the treatment of undifferentiated carcinomas of the thyroid. Langenbecks Arch Surg.

[5] Dixon SJ, Lemberg KM, Lamprecht MR, Skouta R, Zaitsev EM, Gleason CE (2012). Ferroptosis: an iron-dependent form of nonapoptotic cell death. Cell.

[6] Yang WS, Kim KJ, Gaschler MM, Patel M, Shchepinov MS, Stockwell BR (2016). Peroxidation of polyunsaturated fatty acids by lipoxygenases drives ferroptosis. Proc Natl Acad Sci USA.

[7] Xu T, Ding W, Ji X, Ao X, Liu Y, Yu W (2019). Molecular mechanisms of ferroptosis and its role in cancer therapy. J Cell Mol Med.

[8] Chen X, Li J, Kang R, Klionsky D, Tang D (2021). Ferroptosis: machinery and regulation. Autophagy.

[9] Li W, Li S, Higai K, Sasaki T, Asada Y, Ohshima S (2013). Evaluation of licorice flavonoids as protein tyrosine phosphatase 1B inhibitors. Bioorg Med Chem Lett.

[10] Chou TC, Talalay P (1984). Quantitative analysis of dose-effect relationships: the combined effects of multiple drugs or enzyme inhibitors. Adv Enzyme Regul.

[11] Nagai Y, Mimura N, Rizq O, Isshiki Y, Oshima M, Rizk M (2021). The combination of the tubulin binding small molecule PTC596 and proteasome inhibitors suppresses the growth of myeloma cells. Sci Rep.

[12] Deepak AV, Salimath BP (2006). Antiangiogenic and proapoptotic activity of a novel glycoprotein from U. indica is mediated by NF-kappaB and caspase activated DNase in ascites tumor model. Biochimie.

[13] Ha HT, Lee JS, Urba S, Koenig RJ, Sisson J, Giordano T (2010). A phase II study of imatinib in patients with advanced anaplastic thyroid cancer. Thyroid.

[14] Wirth LJ, Eigendorff E, Capdevila J, Paz-Ares LG, Lin CC, Taylor MH (2018). Phase I/II study of spartalizumab (PDR001), an anti-PD1 mAb, in patients with anaplastic thyroid cancer. J Clin Oncol.

[15] Lin B, Lu B, Hsieh IY, Liang Z, Sun Z, Yi Y (2020). Synergy of GSK-J4 with doxorubicin in KRAS-mutant anaplastic thyroid cancer. Front Pharmacol.

[16] Nishimura R, Tabata K, Arakawa M, Ito Y, Kimura Y, Akihisa T (2007). Isobavachalcone, a chalcone constituent of *Angelica keiskei*, induces apoptosis in neuroblastoma. Biol Pharm Bull.

[17] Jing H, Zhou X, Dong X, Cao J, Zhu H, Lou J (2010). Abrogation of Akt signaling by Isobavachalcone contributes to its anti-proliferative effects towards human cancer cells. Cancer Lett.

[18] Jin X, Shi YI (2016). Isobavachalcone induces the apoptosis of gastric cancer cells via inhibition of the Akt and Erk pathways. Exp Ther Med.

[19] Cully M, You H, Levine AJ, Mak TW (2006). Beyond PTEN mutations: the PI3K pathway as an integrator of multiple inputs during tumorigenesis. Nat Rev Cancer.

[20] Wang M, Lin L, Lu JJ, Chen X (2021). Pharmacological review of isobavachalcone, a naturally occurring chalcone. Pharmacol Res.

[21] Manfredi GI, Dicitore A, Gaudenzi G, Caraglia M, Persani L, Vitale G (2015). PI3K/Akt/mTOR signaling in medullary thyroid cancer: a promising molecular target for cancer therapy. Endocrine.

[22] Liu Q, Wang K (2019). The induction of ferroptosis by impairing STAT3/Nrf2/GPx4 signaling enhances the sensitivity of osteosarcoma cells to cisplatin. Cell Biol Int.

[23] Wenz C, Faust D, Linz B, Turmann C, Nikolova T, Dietrich C (2019). Cell-cell contacts protect against t-BuOOH-induced cellular damage and ferroptosis *in vitro*. Arch Toxicol.

[24] Galadari S, Rahman A, Pallichankandy S, Thayyullathil F (2017). Reactive oxygen species and cancer paradox: to promote or to suppress?. Free Radic Biol Med.

[25] Roh JL, Kim EH, Jang HJ, Park YJ, Shin D (2016). Induction of ferroptotic cell death for overcoming cisplatin resistance of head and neck cancer. Cancer Lett.

[26] Yu Y, Xie Y, Cao L, Yang L, Yang M, Lotze MT (2015). The ferroptosis inducer erastin enhances sensitivity of acute myeloid leukemia cells to chemotherapeutic agents. Mol Cell Oncol.

[27] Lu B, Chen XB, Ying MD, He QJ, Cao J, Yang B (2018). The role of ferroptosis in cancer development and treatment response. Front Pharmacol.

